# The effect of lysophosphatidic acid-supplemented culture medium on human immature oocytes matured in vitro

**DOI:** 10.1186/s12958-021-00771-8

**Published:** 2021-06-04

**Authors:** Qigui Xie, Yaxin Xing, Jianhong Zhou, Ling Wang, Jie Wu, Ri-Cheng Chian

**Affiliations:** 1 Department of Obstetrics and Gynecology, Shanghai Tenth People’s Hospital, Nanjing Medical University, Shanghai, China; 2 Center for Reproductive Medicine, Shanghai Tenth People’s Hospital of Tongji University, 301 Yanchang zhonglu, 200072 Shanghai, China; 3 Department of Obstetrics and Gynecology, Jiangsu Province Hospital, The First Affiliated Hospital with Nanjing Medical University, Nanjing Medical University, 300 Guangzhou Road, Nanjing, China

**Keywords:** Oocytes, Cumulus cells, In vitro maturation, Lysophosphatidic acid, Fertility

## Abstract

**Background:**

Lysophosphatidic acid-supplemented culture medium significantly increases the oocyte maturation rate in vitro. However, potential targets and pathways involved remain unknown.

**Methods:**

A total of 43 women, who underwent cesarean section and aged between 18 and 35 years with good health, were included in this study. Immature oocytes were obtained and cultured with 10 µM lysophosphatidic acid. After culture, oocyte maturation was assessed and oocytes and cumulus cells were collected for RNA sequencing. Hierarchical indexing for spliced alignment of transcripts 2 method was used to align clean reads to the human genome. The featureCounts and edgeR package were used to calculate gene expression and analyze differences between groups respectively. ClusterProfiler program was used to perform Gene Ontology and Kyoto Encyclopedia of Genes and Genomes analysis.

**Results:**

Oocyte maturation rate increased significantly following 48 h culture with lysophosphatidic acid. In cumulus cells, Gene Ontology analysis revealed the top 20 items enriched by upregulated genes and downregulated genes respectively; Kyoto Encyclopedia of Genes and Genomes analysis showed that upregulated genes in the treatment group were enriched in TNF signaling and insulin secretion pathways and downregulated genes were enriched in TNF signaling and cell adhesion molecules. In oocytes, Gene Ontology analysis revealed the top 20 items enriched by upregulated genes and downregulated genes respectively; Kyoto Encyclopedia of Genes and Genomes analysis showed that upregulated genes in the treatment group were enriched in MAPK signaling, gap junction, and cell cycle pathways and downregulated genes were enriched in MAPK signaling, estrogen signaling, RAP1 signaling, and gap junction pathways.

**Conclusions:**

Lysophosphatidic acid in culture medium enhances human oocyte maturation in vitro and the identified some potential pathways may associate with oocyte maturation.

## Introduction

The source of oocytes is the key issue for assisted reproductive technologies [1]. There are different ways to obtain in vivo matured oocytes for infertility treatment. Normally, women are given ovarian stimulation to retrieve mature oocytes [2]. However, ovarian stimulations with exogenous gonadotropin are accompanied by direct and indirect side-effects, including life-threatening ovarian hyper-stimulation syndrome. Therefore, mild ovarian stimulation protocols are promoted not only for safety but also their efficiency in developing assisted reproductive technologies [3].

One option to avoid ovarian hyper-stimulation syndrome is to retrieve immature oocytes followed by in vitro maturation (IVM) [4]. Several thousands of healthy babies have been born following IVM for women who were infertile with polycystic ovary syndrome [5]. IVM may also play an important role in fertility preservation for women before cancer treatment, because cell toxic cancer treatments may cause the loss ovarian function [6].

Earlier studies indicated that immature oocytes can be retrieved during cesarean section and the obtained immature oocytes can be matured in vitro [7]. Those immature oocytes may be used for in vitro studies and potentially preserved for fertility preservation [8]. Although studies have reported that the quality of in vitro matured oocytes is affected by different culture media and supplements, it is still unclear how some factors in the culture medium enhance oocyte maturation [9, 10].

Lysophosphatidic acid (LPA) is a membrane phospholipid metabolite with growth factor- and hormone-like effects, and it is present in the human follicular fluid at a concentration of 10–25 µM [11]. Numerous studies have shown that LPA promotes oocyte maturation in vitro, as well fertilization and embryonic development in animals, such as cattle, pig and mouse [12–14]. However, there is no study with LPA in the culture medium on human immature oocytes matured in vitro.

Cumulus–oocyte complex (COC) is the unit that associated with growth and maturation of mammalian oocytes, and is believed to be the site of LPA synthesis and action [15]. In this study, we investigated the effect of LPA in the culture medium on human immature oocyte, and the expression profiles of cumulus cells (CCs) and oocytes. We aimed to reveal important signaling pathways and molecules by which LPA stimulates oocyte maturation in vitro.

## Materials and methods

### Patients

The inclusion criteria were as follows: women who underwent cesarean section in Shanghai Tenth People’s Hospital between November 1st, 2016 and March 31st, 2018; were aged between 18 and 35 years with good health; had not received any medication during pregnancy. Patients were randomly assigned to the control group and LPA treatment group. The enrolled patients were given their informed consent to be included in the study, which received the approval of the Ethics Review Committee of Shanghai Tenth People’s Hospital (No. SHSY-IEC-1.0/16 − 03/01).

### IVM culture media

The control group culture medium comprised of 75 mIU/mL follicle-stimulating hormone (Livzon Co., Zhuhai, China), 75 mIU/mL luteinizing hormone (Livzon Co., Zhuhai, China), and 10 ng/mL human epidermal growth factor (Sigma, St. Louis, MO, USA). The LPA treatment group culture medium contained of 75 mIU/mL follicle-stimulating hormone, 75 mIU/mL luteinizing hormone, 10 ng/mL human epidermal growth factor, and 10 µM LPA (Sigma, St. Louis, MO, USA).

### Acquisition and culture of immature oocytes

Solutions (5 % serum substitute supplement in physiological saline ) used to preserve COCs temporarily were pre-warmed to 37 °C and 1 mL was drawn into 5-ml syringe. At the conclusion of Caesarean Section operation and once the uterus was sutured, the pelvis was wiped clean and dry. The visible antral follicles were aspirated using a 5-mL syringe connected with 20 G needle. The syringes were transferred to the laboratory, and follicular fluid was poured to Petri dishes to look for COCs. COCs with at least three layers of tightly packed CCs with homogeneous oocyte cytoplasm were transferred to culture medium for maturation in culture. COCs from each patient were randomly allocated to one of the two groups: control group and LPA treatment group. COCs were cultured in a two-well dish; the inner well contained 1 mL of IVM medium, and the outer-well contained 2 mL IVM medium. COCs were cultured in an incubator containing 5 % CO_2_ and 5 % O_2_ at 37 °C. Following 24 h of culture, the COCs were denuded from the CCs and assessed for maturity.

For denuding of COCs, they were placed into hyaluronidase solution (80 units/mL) (Irvine Scientific, Santa Ana, CA, USA) for 1 min and pipetted for less than 30 s before COCs were transferred to a petri dish that was contained pre-warmed (37 °C) Sydney IVF Gamete buffer. COCs were pipetted repeatedly to remove the surrounding CCs in order to assess the oocytes maturity. The mature oocyte (metaphase-II) was identified via the extrusion of the first polar body. If they were mature, the oocytes will be vitrified and kept at -196 °C; if they were not mature (GV or MI stage), the immature oocytes will be put back into the incubator for further culture till 48 h and then re-assessed its maturity at the end of culture. Six mature COCs from three women were donated for molecular analysis and the denuded CCs and mature oocytes were collected separately.

### RNA extraction, library preparation, and sequencing

A Smart-Seq2 library was prepared as previously described [16]. Briefly, we performed single-cell sorting, cell lysis, reverse transcription, and cDNA synthesis. The library was constructed using an Illumina Nextera kit and size-selected using AMPure XP beads and examined via quantitative PCR for sequencing. A NovaSeq 6000 System (Illumina, San Diego, CA, USA) was used for sequencing with a PE150 read length. The data were deposited at the National Center for Biotechnology Information with an assigned No. PRJNA678410.

### Expression profile analysis

Clean reads were obtained using the Cutadapt software (http://journal.embnet.org/index.php/embnetjournal/article/view/200) to remove reads containing adaptors and low-quality reads (over 50 % of reads with Q values ≤ 30). HISAT2 was used to align the clean reads to the human genome. The featureCounts program was used to calculate gene expression, and the edgeR package was used to analyze differences between control and LPA group. Genes with *p* < 0.05 and |log2FoldChange| values > 1 were defined as differentially expressed genes.

### Gene ontology (GO) and Kyoto Encyclopedia of Genes and Genomes (KEGG) analysis

The clusterProfiler program was used to perform GO analysis, KEGG analysis and select false discovery rates of < 0.05 to determine pathways enriched by differentially expressed genes (*p* < 0.05).

## Results

### Effect of LPA treatment on human immature oocyte maturation in vitro

**Fig. 1:**
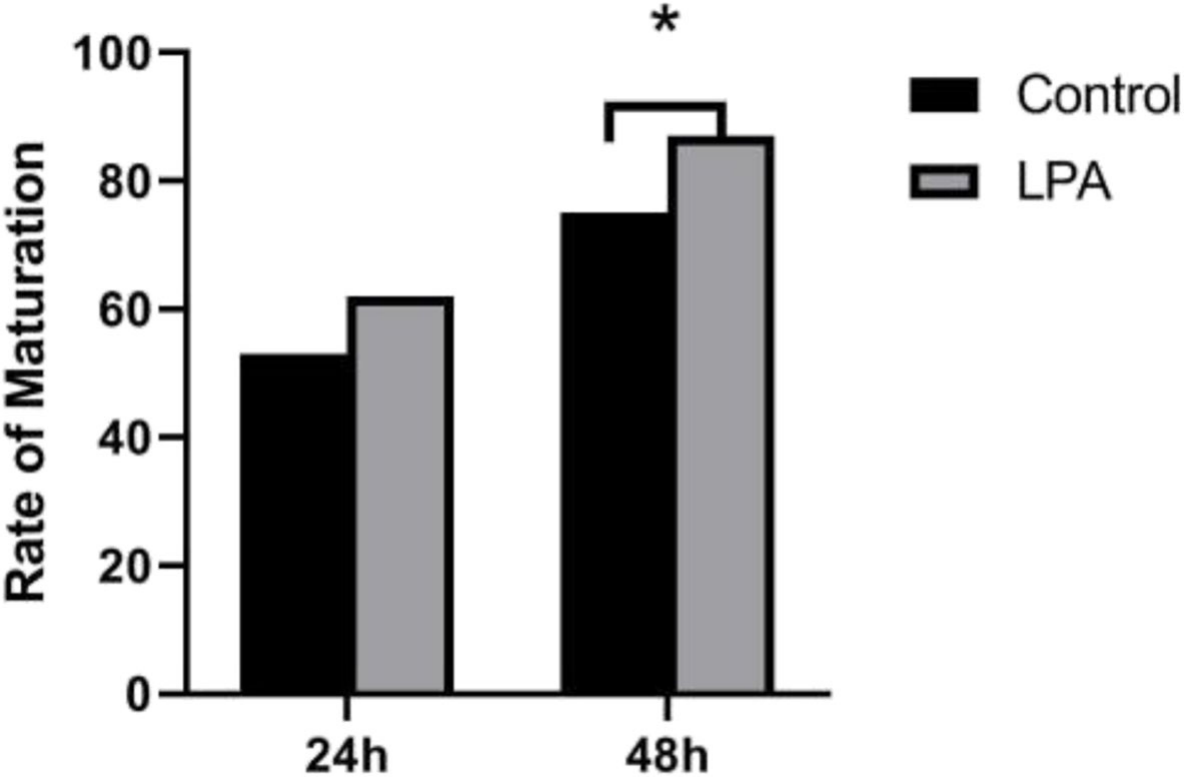
Maturity rate in the control group and LPA group after 24 and 48 h *Indicates significantly different between group (*P* < 0.05). LPA, Lysophosphatidic acid

A total of 155 immature oocytes were obtained. After 48 h of culture, there were 65 out of 74 oocytes in the LPA treatment group and 61 out of 81 oocytes in the control group become mature. The maturation rate was significantly higher (*P* < 0.05) in the LPA treatment group (87.8 %) compared to that of the control group (75.3 %) (Fig. 1).

### LPA treatment altered the expression profiles in the CCs and oocytes

**Fig. 2:**
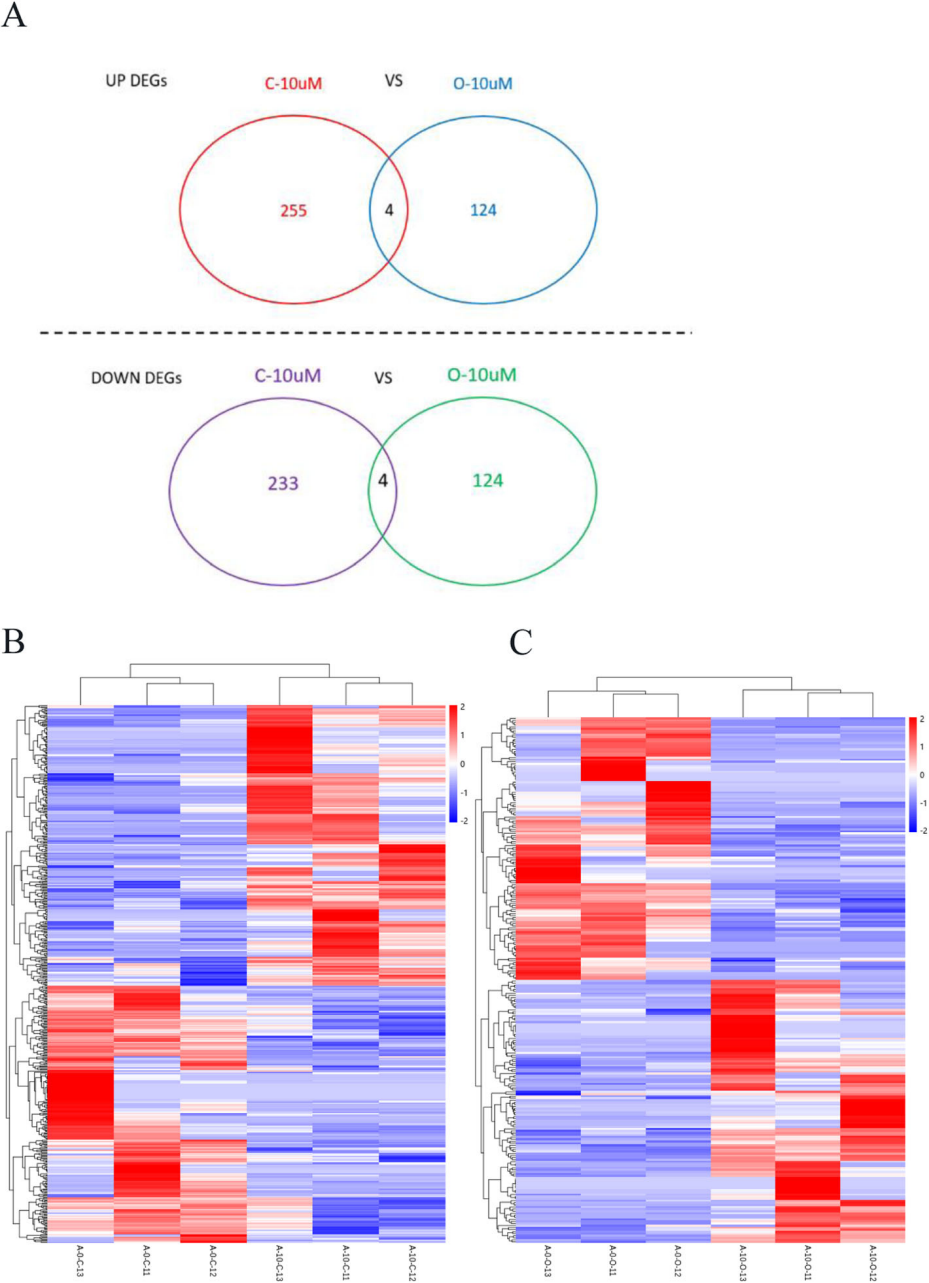
Venn diagram and heatmap of cumulus cells and oocytes between LPA-treated and control groups. **A**) VENN diagrams of differentially expressed genes between control and O-10 µM groups and C-10 µM and control groups. Control group is the group without LPA treatment. O-10 µM group is the group of oocytes treated with LPA at a concentration of 10 µM. C-10 µM group is the group of cumulus cells treated with LPA at a concentration of 10 µM. **B**) Heatmap of differentially expressed genes between C-Control group vs. C-10 µM, in which A-0-C-11, A-0-C-12 and A-0-C-13 were cumulus cells without LPA and A-10-C-11, A-10-C-12 and A-10-C-13 were cumulus cells treated with 10 µM of LPA. **C** Heatmap of differentially expressed genes between O-Control group vs. O-10 µM, in which A-0-O-11, A-0-O-12 and A-0-O-13 were oocytes without LPA and A-10-O-11, A-10-O-12 and A-10-O-13 were oocytes treated with 10 µM of LPA. LPA, Lysophosphatidic acid

There were significant differences in expression profiles in the control and LPA treatment groups. In the CCs, 259 genes were upregulated and 237 genes were downregulated in the LPA treatment group compared to the control group. In the oocytes, 128 genes were upregulated and 128 genes were downregulated in the LPA treatment group compared to the control group. Heatmaps clearly showed that LPA treatment and control groups were classified into different clusters (Fig. 2 A and B).

### LPA treatment led to differences in the enrichment of functional items between cumulus cells and oocytes

**Fig. 3:**
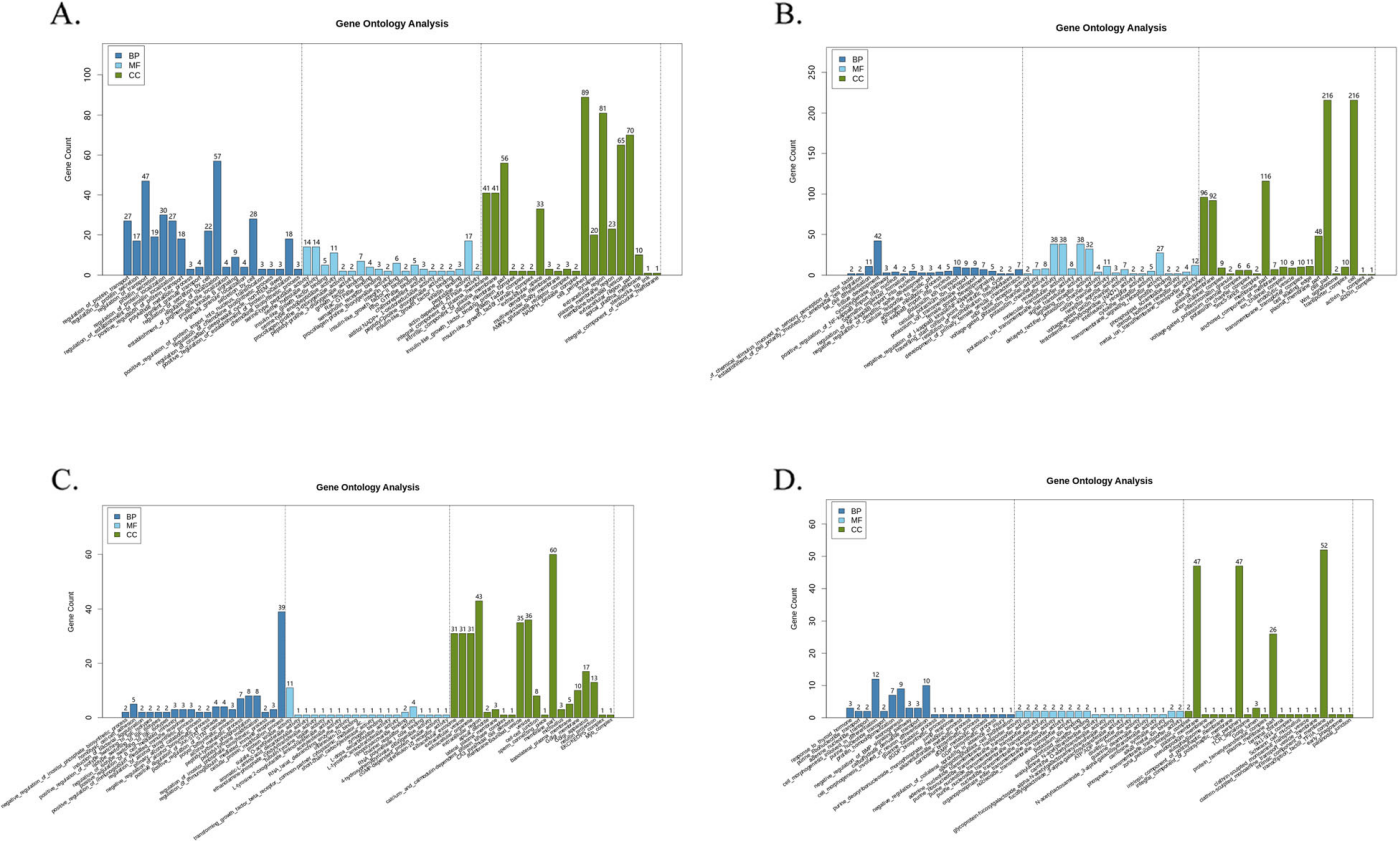
Top20 GO items enriched from differentially expressed genes in the O-10µM and C-10 µM group. O-10 µM group is the oocytes group treated by 10 µM of LPA and C-10 µM group is the cumulus cells treated with 10 µM of LPA. **A**) Top20 GO items enriched from upregulated genes in the C-10 µM group; **B**) Top20 GO items enriched from downregulated genes in the C-10 µM group; **C**) Top20 GO items enriched from upregulated genes in the O-10 µM group; **D**) Top20 GO items enriched from downregulated genes in the O-10 µM group. LPA, Lysophosphatidic acid; GO, Gene Ontology

In the CCs, differentially upregulated and downregulated genes were enriched by GO. The top 20 GO-derived items enriched by upregulated genes that affected BPs were involved in the import, secretion, localization, and transport of proteins and in the biosynthesis and regulation of chemokines, and those that affected the CCs were associated with extracellular substances and components related to the cell periphery and plasma membrane (Fig. 3 A). In contrast, the top 20 GO-derived items enriched by downregulated genes that affected BPs were involved in the mitotic cell cycle and androgen metabolism, and those that affected the CCs were involved in the cell periphery and transmembrane transporter complexes (Fig. 3B).

In oocytes, differentially upregulated and downregulated genes were enriched by GO. The top 20 GO-derived items enriched by upregulated genes that affected BPs were involved in biosynthetic processes and cell-cell adhesion, and those that affected the CCs were involved with the sperm connecting piece and the cell-cell junction (Fig. 3 C). In contrast, the downregulated genes that affected BPs were involved in cell differentiation, dGMP metabolism, and dGDP biosynthesis, and those that affected the CCs were associated with cell periphery (Fig. 3D).

**Fig. 4:**
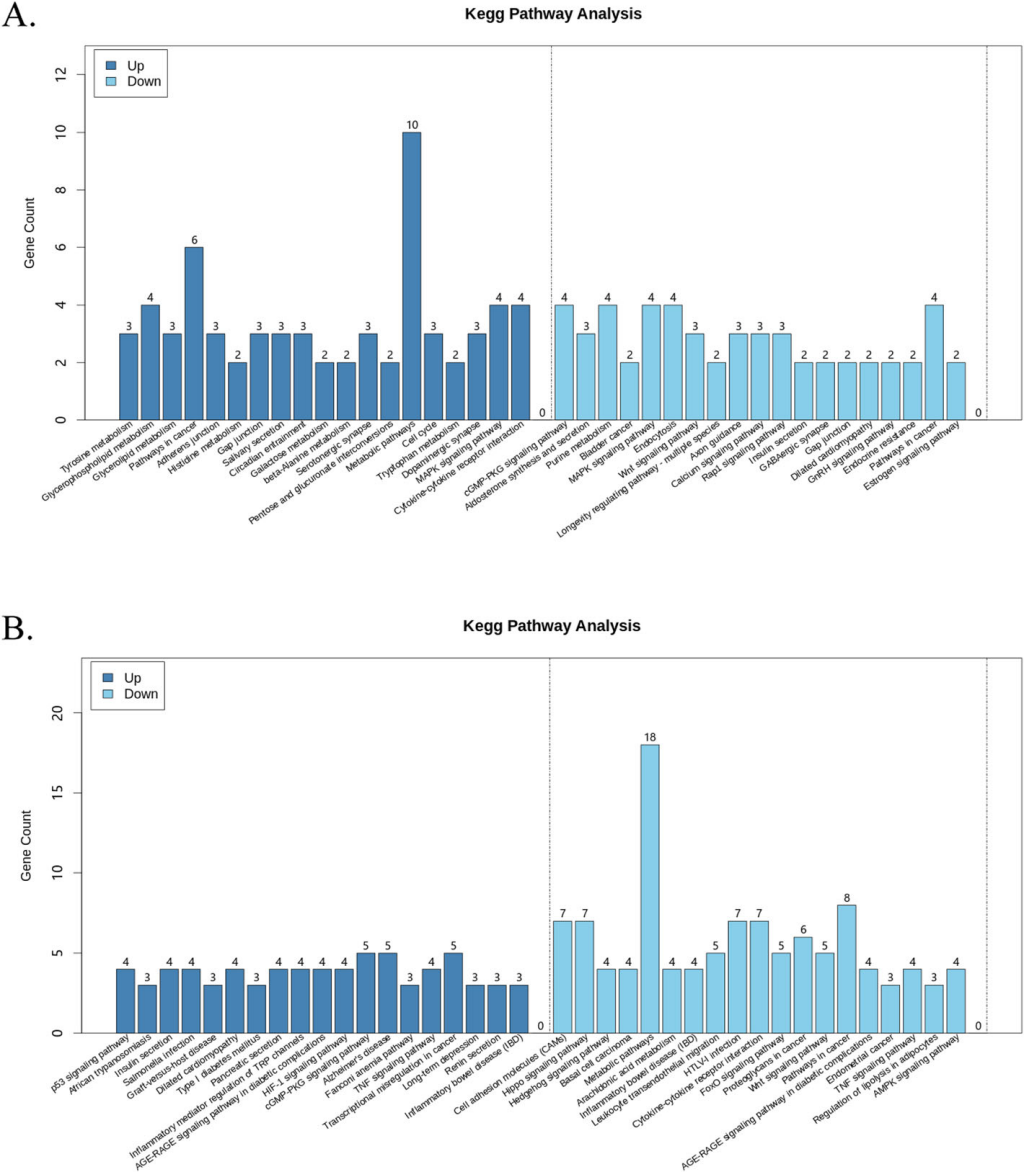
KEGG analysis of differentially expressed genes in the O-10 µM group and C-10 µM group. O-10 µM group is the oocytes treated with 10 µM of LPA and C-10 µM group is the cumulus cells treated with 10 µM of LPA. **A**) KEGG analysis of upregulated and downregulated genes in the C-10 µM group; **B**) KEGG analysis of upregulated and downregulated genes in the O-10 µM group. LPA, Lysophosphatidic acid; KEGG, Kyoto Encyclopedia of Genes and Genomes

KEGG pathway analysis showed that the upregulated genes in the CCs in the LPA treatment group were enriched in TNF signaling and insulin secretion pathways (Fig. 4 A), and pathways enriched by downregulated genes included TNF signaling pathway and cell adhesion molecules pathway (Fig. 4 A). The TNF signaling pathway was enriched by both the upregulation and downregulation of certain differentially expressed genes.

In the oocytes, the upregulated genes in the LPA treatment group were enriched in MAPK signaling, gap junction, and cell cycle pathways (Fig. 4B). The downregulated genes in the LPA treatment were enriched in MAPK signaling, estrogen signaling, RAP1 signaling, and gap junction pathways. MAPK signaling and gap junction pathways were enriched by both upregulated and downregulated genes (Fig. 4B).

## Discussion

We studied the effects of LPA on maturation of human immature oocytes and the changes in the gene expression profiles in the CCs and oocytes during in vitro culture. LPA increases oocyte maturation rate and there were significant differences in the gene expression profile between LPA treatment and control groups.

It has been known that LPA is present in the human follicular fluid at a concentration of 10–25 µM [11]. In our study, we chosed 10 µM of LPA as the final concentration in the culture medium based on previous studied [17]. Studies have shown that *SMAD4* plays an important role during the development of oocytes from small antral follicles (1–3 mm in diameter) to large antral follicles (3–7 mm in diameter) [17]. In a study by Li et al., the litter size was reduced in mice lacking *Smad4* compared to the control mice [18]. In the present study, the expression of this gene was upregulated in oocytes treated with LPA, indicating it may involve in the process of oocyte maturation in vitro.

The biosynthetic function of ribosomes is a critical factor for the development of embryos [19]. In this study, upregulated genes enriched the GO-derived items BP, CC, and MF, which are related to ribosome entry in the LPA-treated cumulus cells. This implies that LPA treatment may stimulate the oocyte maturation in vitro. Interleukin 6 can mediate tyrosine kinase receptor A to regulate cumulus expansion, and interleukin 6 knockout likewise reduces cumulus expansion [20]. Insulin-like growth factor 1 enables CCs to synthesize and accumulate hyaluronic acid, thereby promoting CC expansion [21]. Interleukin 6 and insulin-like growth factor 1 enriched eight items related to the import, secretion, localization, and transport of proteins, as well as the biosynthesis and regulation of chemokines, indicating that interleukin 6 and insulin-like growth factor 1 were key to the promotion of human oocyte maturation by LPA.

Studies by Gebhardt et al. have shown that high levels of PTGS2 expression are related to the rate of live birth [22]. Moreover, high levels of PTGS2 expression in the CCs surrounding mature oocytes are related to high-quality embryos and embryo sacs [23]. In the present study, PTGS2 expression decreased in the CCs of LPA treatment group compared to that in the control group, which suggested that PTGS2 negatively regulated the development of LPA-treated oocytes. However, the specific function of PTGS2 and the mechanism by which it acts require further investigation.

KEGG pathway enrichment analysis revealed upregulated and downregulated genes in the CCs that enrich the TNF signaling pathway, and the CCs can release soluble TNF-α to promote oocyte aging [24]. In our study, the upregulated genes in the CCs enriched the insulin secretion pathway. Insulin plays a central role in polycystic ovary syndrome, and it engages with the insulin-like growth factor 1 receptor to enhance steroid production in ovaries and adrenal glands [25]. Downregulated differentially expressed genes enrich signaling pathways related to cell adhesion molecules and AMPK. In the CCs, the downregulation of cell adhesion molecules may cause polycystic ovary syndrome [26].

Among the top 20 GO-derived items enriched by upregulated genes in the oocytes of the LPA-treated group, five were related to biosynthetic processes and one was related to cell-cell adhesion. Both *PLEK* and *PRKG1* enriched these six items. The expression of PLEK may promote cell growth and development [27], and PRKG1 may be related to early life adversity [28]. The present results suggested that PLEK and PRKG1 were important for the maturation and development of oocytes in the culture medium supplemented with LPA, but there have not been any reports on these two genes regarding human oocyte maturation. We found that 47 downregulated genes enriched CC items related to the cell periphery; it is interesting to note that chromosomes separate during cell division at the cell periphery [29]. Thus, our results indicated that LPA may affect cell division.

In the oocytes, both upregulated and downregulated genes enriched items related to the MAPK signaling pathway and the gap junction. MAPK signaling pathway regulates the development of oocytes [30]. During the development of COCs, the gap junction pathway mediates material exchange between oocytes and CCs [31]. Downregulated genes enriched the estrogen signaling pathway and the RAP1 signaling pathway. Estrogen affects the physiological development of women and plays an important role in the maturation of oocytes [32]. The RAP1 signaling pathway in oocytes involves multiple cellular processes, including secretion, cell adhesion, and intercellular junction formation, and regulates oocyte maturation and embryonic development [33].

Lysophosphatidic acid receptor (LPAR) is a G-protein coupled receptor family, which includes LPAR1, LPAR2, LPAR3, LPAR4, LPAR5 and LPAR6. Since LPA exercises its actions via LPARs, we further investigated the expression of LPARs. LPAR1, LPAR4, LPAR5 and LPAR6 were upregulated while LPAR2 was downregulated in the CCs. LPAR1 and LPAR6 were upregulated in ooctyes while LPAR2 and LPAR5 were downregulated in oocytes. The results implied that LPAR4 might play a role in LPA-stimulated the CCs but not in LPA-stimulated oocytes. LPARs’ expression pattern in our study was different from that in mouse [34]. Their exact roles in LPA-stimulated COCs deserve further studies.

There are limits in our study. Due to the limited number of participants and available immature oocytes, we had a relatively small number of the CCs and oocytes for RNA sequencing. If possible in clinics, we will increase the sample size in future studies.

## Conclusions

Oocyte maturation is promoted by LPA in the culture medium and identified some potential targets and pathways associated with oocyte maturation in vitro. Further study is required to understand its mechanism in order to apply for clinical significance.

## Data Availability

The datasets supporting the conclusions of this article are available in the National Center for Biotechnology Information repository, No. PRJNA678410 and hyperlink to datasets in https://www.ncbi.nlm.nih.gov/bioproject/PRJNA678410.

## Abbreviations

IVM: In vitro maturation
LPA: Lysophosphatidic acid
COC: Cumulus–oocyte complex
GO: Gene ontology
KEGG: Kyoto Encyclopedia of Genes and Genomes
BP: Biological process
CC: Cellular component
MF: Molecular function
